# Parenting sense of competence among chinese parents of premature infants: a cross-sectional study

**DOI:** 10.1186/s12884-023-05703-5

**Published:** 2023-05-30

**Authors:** Long Huang, Xiao-juan Wang, Gui-hua Liu, Xiao-ting Li, Yu-hong Zhang, Bing-yue Zhao, Rong-fang Hu

**Affiliations:** 1 The School of Nursing, Fujian Medical University, Fuzhou, 350122 China; 2 Fujian Maternity and Child Health Hospital, College of Clinical Medicine for Obstetrics & Gynecology and Pediatrics, Fujian Medical University, Fuzhou, 350000 China; 3 School of Nursing, Fujian Medical University, 1 Xue Yuan Road, University Town, Fuzhou, Fujian China

**Keywords:** Competence, Premature birth, Parents, Social support, Depression, Cross-sectional study

## Abstract

**Background:**

Parenting sense of competence is not only indispensable to the wellbeing of the parents of premature infants, but is also pivotal to the overall development of these infants. This study examined the level of parenting sense of competence and its associated factors in Chinese parents of preterm infants.

**Methods:**

This cross-sectional study was performed at a university teaching hospital in Fuzhou (China) from December 2021 to April 2022. Data were collected using the Parenting Sense of Competence Scale, Edinburgh Postnatal Depression Scale, Social Support Rating Scale, Parenting Care Knowledge Subscale, Parenting Care Skill Subscale, and a sociodemographic questionnaire.

**Results:**

A total of 401 Chinese parents were included in the analysis. The average parenting sense of competence scale score was 70.93 ± 13.06. After controlling for demographic characteristics, parenting knowledge (*β* = 0.149, *P* = 0.013), parenting skills (*β* = 0.241, *P* < 0.001), social support (*β* = 0.184, *P* < 0.001) and depression (*β* = −0.272, *P* < 0.001), were significantly associated with the parenting sense of competence score, and explained 43.60% of the variance in this score.

**Conclusions:**

Chinese parents of preterm infants were found to have a moderate parenting sense of competence. This could be further improved through efforts aimed at reducing depressive symptoms and increasing parenting knowledge, parenting skills, and social support.

## Background

With advancements in preterm infant care, the survival rate of vulnerable preterm infants has improved significantly over the past few decades in China [1]. However, compared with healthy full-term infants, surviving preterm infants are more likely to experience a heightened risk of short- and long-term impairments in neurological, sensory, cognitive, and behavioral development [2]. Research widely supports parenting quality as a critical social factor for optimizing the overall development of preterm infants [3]; an important predictor of the parenting quality is parenting sense of competence [4].

Parenting sense of competence refers to the parents’ personal evaluation of their capacity to care for their infants (usually conceptualized as perceived self-efficacy in parenting) as well as their contentment with their parenting role [5]. Acquisition of parenting sense of competence is increasingly being considered a modifiable determinant of parental role adaptation; this has significant for improvement of parenting quality as well as the cognitive and social improvement of preterm infants [6]. Parents with a low level of parenting sense of competence are more prone to feeling disinterested in interacting with their vulnerable infants; this may hamper the parents’ opportunities for bonding with their child and hinder their transitioning to parenthood [7]. Conversely, a strong parenting sense of competence has been shown to foster positive childrearing practices and parent-infant attachment [8]. Therefore, comprehensively understanding parenting sense of competence and its associated variables, and accordingly devising effective interventions for improving the overall development of preterm infants is crucial.

Most studies on parenting sense of competence have focused on parents of full-term infants or infants with specific problems, such as, autism, multiple sclerosis, and intellectual disability [9–11]. Only few studies have investigated parenting sense of competence among parents of preterm infants [12]. Furthermore, these studies have certain limitations, for instance, they have small sample sizes, samples comprising mothers alone or focused on investigations performed only during hospitalization and at early discharge. For example, Koliouli et al. found that parenting sense of competence was worse among fathers of preterm infants exhibited worse sense of than among those of full-term infants; however, their study comprised 48 fathers [13]. Furthermore, a cross-sectional survey revealed that age, monthly per capita household income, and parenting experience influenced parenting sense of competence [14]. However, Duan et al. reported that monthly per capita household income did not affect parenting sense of competence among mothers of preterm infants [15].

Evidence from other populations (e.g., parents of healthy infants or parents of children with “special” diseases) has shown that social support, depression, and family functioning are also associated with parenting sense of competence [16–18]. However, to our knowledge, related studies on parents of preterm infants have primarily focused on the relationship between parenting sense of competence and depression or social support. For instance, Wu et al. found a significant negative correlation between depressive symptoms and parenting sense of competence among preterm primiparous mothers at 3 months postpartum [19]. Furthermore, some studies have demonstrated that augmenting parental knowledge and skills could improve parenting sense of competence [20, 21]; however, the associations among these factors have not been studied extensively. Therefore, we performed this cross-sectional study, to investigate the level of parenting sense of competence and its relationship with the above-mentioned factors among parents of preterm infants. We hypothesized that parenting sense of competence in these parents is related to a various demographic (e.g., gestational age and education level), psychological (depressive symptoms), and sociocultural (social support and family functioning) factors. Accordingly, our study had the following three objectives:

1. To establish the level of parenting sense of competence among parents of preterm infants within the first year after preterm birth.
2. To evaluate whether the sociodemographic characteristics of preterm infants’ and their parents’ are associated with parenting sense of competence.
3. To examine the relationships among depressive symptoms, social support, family functioning, parenting knowledge, parenting skill, and parenting sense of competence.

## Methods

### Study design, setting, and participants

This cross-sectional study was performed between December 2021 and April 2022 at a university teaching hospital in Fuzhou (capital of the Fujian Province, southeast China). Fuzhou has a population of approximately 8.42 million, and this hospital records more than 3,000 births annually.

The study sample comprised parents of preterm infants. The inclusion criteria were as follows: (1) age > 18 years, (2) ability to read and complete the questionnaires, (3) having infants born between 25 and 37 weeks of gestation, and aged less than 1 year at the time of inclusion. The exclusion criteria were as follows: (1) presence of cognitive dysfunction, or a family history of psychiatric illness and (2) having premature infants diagnosed with genetic abnormalities, cerebral palsy, or a congenital disability (e.g., cleft lip and palate or deafness).

### Sample

The sample size was calculated using the Power Analysis and Sample Size software. With alpha set to 0.05, a sample size of 395 was found to have 80% power for detecting an effect size (f^2^) of 0.02 in multiple regression analyses. Accordingly, the 401 parents included in the final study sample provided sufficient power or detecting minimum effect sizes.

### Measurement

Parenting sense of competence were measured using the Chinese version of the Parenting Sense of Competence Scale (C-PSOC), C-PSOC comprises 17 items that are divided into the perceived parental role competence subscale (PSOC-E; eight-items) and the parenting satisfaction subscale (PSOC-S; nine-items). Each item is scored on a 6-point Likert scale, with scores ranging from 1 (strongly disagree) to 6 (strongly agree). The scores of all items are summed to obtain the overall PSOC score (range: 17–102). The score ranges for PSOC-E and PSOC-S are 8 to 48 and 9 to 54, respectively. A higher score indicates a higher competence and parenting satisfaction. In a previous study, the Cronbach’s alpha was 0.87 and the internal consistency ranged from 0.78 to 0.88 [6]. In this study, the Cronbach’s alpha was 0.93.

Parental depressive symptoms were evaluated using the Edinburgh Postnatal Depression Scale (EPDS). The EPDS is the most popular instrument for screening depression throughout the perinatal period [22]. It comprises 10 items, and each item is from 0 to 3; thus, the overall score ranges from 0 to 30, with higher scores indicating more severe depressive symptoms. Cut-off scores of 10 and 13 have been recommended to indicate high levels of depressive symptoms in fathers and mothers, respectively. In a previous study, the Cronbach’s alpha was 0.78, and the test retest reliability was 0.90 [23]. In the present study, the Cronbach’s alpha was 0.86.

Social support was measured using the Social Support Rating Scale (SSRS); this instrument is commonly used to assess an individual’s personal social support within Chinese communities [24]. It is a 10-item, three-dimensional self-reporting instrument that assesses subjective support, objective support, and support utilization. Seven items are rated on a 4-point Likert scale, with scores ranging from 1 to 4 ; the remaining three items are rated by calculating the number of support sources. Higher scores indicate a greater level of social support. The SSRS has exhibited high reliability and validity for use among Chinese parents in a previous study, with Cronbach’s alpha values of 0.73 and 0.75 for Chinese mothers and fathers, respectively [25]. In the present study, the Cronbach’s alpha value was 0.83.

Family functioning was evaluated using the Family “Adaptability, Partnership, Growth, Affection, and Resolve” Index (APGAR) (APGAR). Compiled by Smilkstain [26], it is a self-administered questionnaire that assesses an individual’s satisfaction with their family functioning. The instrument comprises five questions, one each on adaptation, partnership, growth, affection, and resolution; the response to each is scored on a 3-point Likert scale (0 = hardly ever, 1 = occasionally, and 2 = almost always). The overall scores range from 0 to 10; scores of 8–10, 4–7, 0–3 suggest positive, moderate family dysfunction, severe family dysfunction, respectively. In a previous study, the Cronbach’s alpha was 0.87 [27]; in this study, the Cronbach’s alpha was 0.89.

Parenting knowledge and skills were evaluated using the care knowledge and care skills subscales of the Premature Infant Care Competence Scale for Parents in transition (PICCS), respectively. Developed by Zhao in 2020 [28]; the complete PICCS is a 35-item instrument, and each item is rated on a 5-point Likert scale. The care knowledge and care skills subscales comprise 13 and 11 items, respectively; these are related to common knowledge and skills pertaining to the daily care of premature infants, respectively. Each item is rated from 1 (completely know) to 5 (completely do not know), with higher scores indicating a higher level of parenting knowledge and skills. In our study, the Cronbach’s alpha coefficients for the care knowledge and care skills subscales were 0.95 and 0.94, respectively.

Using a sociodemographic data sheet, the following parental data were obtained: age, occupation status, educational level, and monthly household income (per person). Furthermore, the infants’ data (e.g., sex and age) were also collected.

### Data collection and procedures

The data were collected by two trained researchers (L.H and BY.Z) at a preterm infant follow-up clinic. To ensure an accurate and consistent data collection process, the researchers explained the survey’s purpose and procedure to the parents once they met the inclusion criteria and assured them that all data would be kept confidential. After receiving written informed consent, the parents were invited to complete the five self-reporting instruments (which required approximately 15–20 min).

### Statistical analysis

Data were analyzed using IBM SPSS Statistics 26.0 (version 26.0, IBM Corp, Armonk, NY, Stata Corp U.S.A.). Descriptive statistics were computed to describe parental characteristics. Since our data were non-normally distribute of our data, non-parametric tests were used to compare PSOC score, and parental sociodemographic characteristics. Spearman correlation analyses were performed to explore the relationships between the PSOC score and continuous variables. A multivariate linear regression was performed as follows to identify variables that were independently associated with the PSOC score (*P* < 0.05). In Model 1, PSOC score was considered the dependent variable, while significant sociodemographic factors (*P <* 0.15 in the univariate analysis) were considered the independent variables. In Model 2, parenting knowledge, parenting skills, social support, family functioning, and depression were analyzed as predictors. Finally, Model 3 was adjusted to control for potential confounders (e.g., gender, monthly income per capita, and education degree).

## Results

### Demographic characteristics of the participants

Throughout the recruitment period, 452 eligible parents were contacted; among these, 51 parents (11.3%) declined participation for personal reasons (e.g., lack of time). The remaining, 401 parents completed the survey (response rate of 88.7%). Among these, 184 (45.90%) were fathers and 217 (54.10%) were mothers. The mean age of the parents was 31.93 years (range 20–46 years, standard deviation [SD] = 4.61), whereas the mean age of their non-participating spouses was 32.28 years (range: 18–49 years, SD = 4.81). Most parents and their non-participating spouses had obtained at least a high school degree (parents: 82.00%, spouses: 82.5%); furthermore, most were public officials (parents: 59.40%, spouses: 70.00%). Most preterm infants were in the least serious preterm category, with most categorized as having a moderate/late gestational level (gestational age: 32–36 weeks). The detailed demographic characteristics of the participants are presented in Table 1.

**Table 1:** Demographic characteristics of participants (n = 401)

Variables	N (%)	Medium (Q1, Q3)	P
Gender			
Male	184 (45.90)	69.00 (64.00, 81.00)	Z = -2.069, *P* = 0.039^*^
female	217 (54.10)	67.00 (61.00, 77.00)	
**Baby gender**			
boy	237 (59.10)	68.00 (61.00, 80.00)	Z = -0.583, *P* = 0.560
girl	164 (40.90)	69.00 (63.00, 78.00)	
**Multiple Birth**			
No	311 (77.60)	68.00 (62.00, 77.00)	Z = -1.259, *P* = 0.208
Yes	90 (22.40)	69.50 (61.75, 86.00)	
**Type of delivery**			
Normal vaginal delivery (NVD)	176 (43.90)	68.00 (61.00, 76.75)	Z = -0.563, *P* = 0.574
Cesarean section	225 (56.10)	69.00 (62.00, 80.50)	
**Gestation age at birth**			
< 24 to < 34	172 (42.90)	67.00 (60.25, 77.00)	Z = -1.836, *P* = 0.066
≥ 34 to < 37	229 (57.10)	69.00 (63.00, 81.00)	
**Time since discharge (d)** ^**a**^			
≤ 7	104 (25.90)	68.50 (63.00, 77.75)	H = 9.496, *P* = 0.023*
>7 to ≤ 20	98 (24.40)	67.50 (62.00, 82.50)	
>20 to ≤ 40	105 (26.20)	72.00 (62.00,82.50)	
>40	94 (23.40)	66.00 (59.00,73.00)	
**Length of hospital stay (d)** ^**a**^			
≤ 7	124 (30.90)	68.00 (62.00, 81.00)	H = 1.989, *P* = 0.575
>7 to ≤ 14	115 (28.70)	70.00 (62.00, 79.00)	
>14 to ≤ 30	75 (18.70)	67.00 (60.00, 77.00)	
>30	87 (21.70)	67.00 (62.00, 77.00)	
**Birth weight (g)**			
< 1500	80 (20.00)	67.50 (63.00, 79.75)	H = 0.309, *P* = 0.857
≥ 1500 to < 2500	227 (56.60)	68.00 (62.00, 77.00)	
≥ 2500	94 (23.40)	69.00 (60.75, 81.00)	
**Mode of conception**			
Natural conception	338 (84.30)	68.00 (61.75, 78.00)	Z = -1.082, *P* = 0.279
Other ^**b**^	63 (15.70)	69.00 (63.00, 87.00)	
**Infant feeding pattern**			
Exclusive breastfeeding	154 (38.40)	70.00 (63.00, 81.00)	H = 5.178, *P* = 0.075
Partial breastfeeding	168 (41.90)	68.00 (62.00, 77.00)	
Bottle feeding	79 (19.70)	65.00 (60.00, 79.00)	
**Family per capita monthly income (CNY)**			
≤ 3000	76 (19.00)	66.00 (61.00, 72.00)	H = 14.224, *P* = 0.003^**^
>3000 to ≤ 5000	151 (37.70)	67.00 (61.00, 78.00)	
>5000 to ≤ 8000	124 (30.90)	68.50 (63.00, 84.75)	
>8000	50 (12.50)	74.50 (67.00, 84.00)	
**Family type**			
nuclear family	58 (14.50)	72.00 (65.00, 78.25)	Z = -1.339, *P* = 0.181
Extended family	343 (85.50)	68.00 (61.00, 79.00)	
**First time parent**			
Yes	243 (60.60)	68.00 (61.00, 78.00)	Z = -0.457, *P* = 0.648
NO	158 (39.40)	68.50 (62.00, 79.00)	
**Age**			
≤ 25	27 (6.70)	64.00 (59.00, 70.00)	H = 4.856, *P* = 0.183
>25 to ≤ 30	127 (31.70)	67.00 (61.00, 85.00)	
>30 to ≤ 35	163 (40.60)	69.00 (62.00, 78.00)	
>35	84 (20.90)	69.50 (64.00, 80.75)	
**Education degree**			
Junior middle school or below	72 (18.0)	66.00 (61.00, 71.75)	H = 8.796, *P* = 0.032^*^
High school or technical secondary school	77 (19.20)	67.00 (62.00, 74.50)	
Tertiary	83 (20.70)	68.00 (61.00, 80.00)	
university or above	169 (42.10)	71.00 (62.50, 83.00)	
**Occupational status** ^c^			
Non-public officials	163 (40.60)	67.00 (61.00, 74.00)	Z = -1.915, *P* = 0.056
Public officials	238 (59.40)	69.00 (62.00, 81.00)	
**Parenting experience**			
No	256 (63.80)	68.00 (61.00, 78.00)	Z= -0.297, *P* = 0.767
Yes	145 (36.20)	68.00 (62.00, 79.00)	
**Spouse’s age**			
≤ 25	90 (22.40)	67.00 (60.00, 77.25)	H = 3.416, *P* = 0.332
>25 to ≤ 30	66 (16.50)	67.50 (64.00, 79.00)	
>30 to ≤ 35	154 (38.40)	68.00 (61.00, 78.00)	
>35	91 (22.70)	71.00 (64.00, 80.00)	
**Spouse’s education degree**			
Junior middle school or below	70 (17.50)	66.00 (61.75, 72.00)	H = 17.388, P < 0.001^***^
High school or technical secondary school	84 (20.90)	65.50 (59.25, 71.75)	
Tertiary	88 (21.90)	67.50 (62.00, 80.75)	
university or above	159 (39.70)	72.00 (64.00, 82.00)	
**Spouse’s occupational status**			
Non-public officials c	168 (41.90)	67.00 (61.00, 73.00)	Z = -2.782, *P* = 0. 005^**^
Public officials	233 (58.10)	70.00 (63.00, 81.00)	
**Spouse’s parenting experience**			
No	254 (63.30)	68.00 (61.00, 78.00)	Z = -0.417, *P* = 0. 677
Yes	147 (36.70)	68.00 (62.00, 79.00)	

Note: a = Grouping according to interquartile spacing; b = Others include artificial insemination or in vitro fertilization; c = Public officials include teacher, civil servant, office staff and Non-public officials include Self-employed, Freelance; Z /H = Mann-Whitney Z test / Kruskal-Wallis H test; * *P* < 0.05, ** *P* < 0.01, *** *P* < 0.001

### Level of parenting sense of competence

The descriptive statistics of the parenting sense of competence are presented in Table 2. The mean PSOC score was 70.93 (SD = 13.06; range: 40.00-102.00). The mean PSOC-E score was 34.57 (SD = 6.58; range: 18.00–48.00), whereas, the mean PSOC-S score was 36.36 (SD = 8.20; range:16.00–54.00).

**Table 2:** Level of parenting sense of competence (n = 401)

Variables	Medium (Q1, Q3)	Range (Min, Max)	Mean (SD)
**PSOC total score**	68.00 (62.00, 78.50)	(40.00, 102.00)	70.93 (13.06)
parenting efficacy (PSOC Efficacy subscale)	33.00 (30.50, 38.00)	(18.00, 48.00)	34.57 (6.58)
parenting satisfaction (PSOC Satisfaction subscale)	35.00 (31.00, 41.00)	(16.00, 54.00)	36.36 (8.20)

Note: SD = standard deviations; PSOC = parenting sense of competence scale

### Correlations among the scores for PSOC, SSRS, parenting knowledge and skills, and APGAR

As shown in Table 3, the scores for SSRS, APGAR, the parenting knowledge subscale, and the parenting skills subscale were significantly and positively correlated with the PSOC score (*P* < 0.001). However, the EPDS scores were significantly negatively correlated with the PSOC scores (*r* = -0.484, *P* < 0.001).

**Table 3:** Correlations between parenting sense of competence and the key variables (n = 401)

Variable	Mean	SD	parenting sense of competence	depression	family functioning	social support	parenting knowledge	parenting skills
**parenting sense of competence**	70.93	13.06	1.000					
**depression**	8.39	5.21	-0.484^***^	1.000				
**family functioning**	7.54	2.61	0.288^***^	-0.295^***^	1.000			
**social support**	43.50	7.91	0.407^***^	-0.428^***^	0.369^***^	1.000		
**parenting knowledge**	42.65	9.56	0.445^***^	-0.307^***^	0.371^***^	0.371^***^	1.000	
**parenting skills**	40.83	7.42	0.420^***^	-0.270^***^	0.301^***^	0.301^***^	0.744^***^	1.000

Note: SD = standard deviations; ***P < 0.001

### Factors associated with the PSOC scores

Univariate analysis revealed that gender (*P* = 0.039), time since discharge (*P* = 0.023), monthly income per capita (*P* = 0.003), education degree (*P* = 0.033), spouse’s education degree (*P* = 0.033), and spouse’s occupational status (*P* = 0.005) were significantly associated with parenting sense of competence (see Table 1).

The results of the multiple regression analysis are shown in Table 4. In Model 1, gender, time since discharge, monthly income per capita, education degree, spouse’s education degree, and spouse’s occupational status together accounted for 7.20% of the variance in the PSOC score. In Model 2, depression (*β* = −0.295, *P* < 0.001), social support (*β* = 0.202, *P* < 0.001), parenting knowledge (*β* = 0.150, *P* = 0.014), and parenting skills (*β* = 0.212, *P* < 0.001) were significantly associated with the PSOC score; the model explained 39.40% of the variance in this score. Finally, in Model 3, (after controlling for demographic characteristics), depression (*β* = −0.272, *P* < 0.001), social support (*β* = 0.184, *P* < 0.001), parenting knowledge (*β* = 0.149, *P* = 0.013) and parenting skills (*β* = 0.241, *P* < 0.001) were significantly associated with the PSOC score; the model explained 43.60% of the variance in this score. Moreover, there was no multicollinearity in the independent variables in any of the three regression models.

**Table 4:** Multiple linear regression analysis predicting parenting sense of competence (N = 401)

Variable		Model 1			Model 2			Model 3		
		Beta	P	VIF	Beta	P	VIF	Beta	P	VIF
**Gender**	Male	Ref	-	-	-	-	-	Ref	-	-
	female	-0.068	0.191	1.118	-	-	-	-0.043	0.315	1.254
**Time since discharge (d)**	≤ 7	Ref	-	-	-	-	-	Ref	-	-
	>7 to ≤ 20	0.041	0.499	1.530	-	-	-	0.042	0.376	1.541
	>20 to ≤ 40	0.070	0.253	1.541	-	-	-	0.025	0.604	1.605
	>40	-0.061	0.318	1.531	-	-	-	-0.044	0.364	1.587
**Family per capita monthly income (CNY)**	≤ 3000	Ref	-	-	-	-	-	Ref	-	-
	>3000 to ≤ 5000	0.054	0.460	2.247	-	-	-	0.011	0.848	2.283
	>5000 to ≤ 8000	0.142	0.060	2.344	-	-	-	0.053	0.374	2.398
	>8000	0.149	0.027*	1.879	-	-	-	0.107	0.044*	1.904
**Education degree**	Junior middle school or below	Ref	-	-	-	-	-	Ref	-	-
	High school or technical secondary school	0.043	0.540	2.012	-	-	-	-0.007	0.892	2.038
	Tertiary	0.066	0.385	2.365	-	-	-	0.074	0.211	2.377
	university or above	0.071	0.440	3.482	-	-	-	0.066	0.365	3.520
**Spouse’s education degree**	Junior middle school or below	Ref	-	-	-	-	-	Ref	-	-
	High school or technical secondary school	-0.048	0.501	2.097	-	-	-	-0.079	0.157	2.121
	Tertiary	0.022	0.796	2.916	-	-	-	0.010	0.883	2.940
	university or above	0.092	0.378	4.469	-	-	-	0.034	0.678	4.501
**Spouse’s occupational status**	Non-public officials	Ref	-	-	-	-	-	Ref		
	Public officials	-0.049	0.509	2.256	-	-	-	-0.024	0.683	2.260
**Depression**	-	-	-	-	-0.295	<0.001	1.279	-0.272	<0.001	1.422
**Family functioning**	-	-	-	-	-0.032	0.459	1.207	-0.050	0.250	1.281
**Social support**	-	-	-	-	0.202	<0.001	1.432	0.184	<0.001	1.501
**Parenting knowledge**	-	-	-	-	0.150	0.014*	2.383	0.149	0.013*	2.418
**Parenting skills**	-	-	-	-	0.212	<0.001	2.252	0.241	<0.001	2.428

Note: Model 1: R^2^ = 0.072, Adjusted R^2^ = 0.038, F = 2.143, p = 0.009;

Model 2: R^2^ = 0.394, Adjusted R^2^ = 0.386, F = 51.397, p < 0.001;

Model 3: R^2^ = 0.436, Adjusted R^2^ = 0.408, F = 15.488, p < 0.001;

* *P* < 0.05, ** *P* < 0.01

## Discussion

To the best of our knowledge, this study is the first to investigate the level and predictors of parenting sense of competence among parents of preterm infants within the first year of preterm birth in China. We found that parents of premature infants showed a moderate parenting sense of competence. We also found that of all demographic variables, only the monthly household income (per person) was a significant predictor of parenting sense of competence among parents of preterm infants. Further, a core finding of this study was that depression, social support, parenting knowledge, and parenting skills were the key factors for parenting sense of competence, and that these variables collectively contributed to 43.6% of the variance in the PSOC score. This is a new insight into clinical care practice; compared with relying on one of these variables, a comprehensive assessment of social support, depression, parenting knowledge, and parenting skills may be more accurate and useful.

Our findings revealed that these parents exhibited a moderate parenting sense of competence; the PSOC score obtained in our study (70.93 ± 13.06) is lower than that obtained in a recent study on Chinese parents of healthy full-term infants [29]. In another study, the mean PSOC score for Spanish parents of full-term infants was 77.77 [10], which is also higher than obtained in this study. Compared with full-term infants, most preterm infants are typically admitted to the neonatal intensive care immediately at birth. This interruption in the development of a healthy parent-infant attachment, along with prognostic uncertainty and a prolonged separation between the parents and their infants, may lead to a decline in parenting sense of competence [13]. Notably, the level of overall parenting sense of competence in this study was significantly higher than that in Wu et al. in China (mean maternal PSOC score: 59.32), although both studies had the same study design and involved the same measurement tool [19]. This significant difference may be explained by the differing study populations. Wu et al. investigated primiparous mothers of preterm infants at 3 months postpartum, while we investigated parents of preterm infants. Owing to inadequate childcare experience, first-time mothers of preterm infants are often less likely to feel competent and fulfilled in their role as mothers [18].

Contrary to our expectations, no associations were observed between parenting sense of competence and the sociodemographic characteristics of preterm infants. Although the PSOC score of parents of late preterm infants (born after 34 week gestational weeks) were higher than those of parents of moderately, very, or extremely preterm infants, this difference was not statistically significant (*P* = 0.066). Pennell C et al. also obtained similar results; they discovered that the prematurity level did not influence parenting sense of competence [30]. Another study similarly revealed that the occurrence of a preterm birth itself is the main influencing factor for maternal sense of competence [31]. Our findings may be explained by the fact that we evaluated parenting sense of competence during the first year postpartum, and thus, parents of both very preterm and late preterm infants had adequate opportunities to master their parenting skills, and build a sufficient sense of parenting competence within the first few months following birth.

In accordance with a previous study [14], our study revealed that out of all parental sociodemographic factors, the monthly household income (per person) was retained in the final regression model, and was thus, a significant predictor of parenting sense of competence. The average first-year cost of raising a preterm infant is three times higher than that of raising a healthy full-term infant [32]. However, a previous study revealed that only 50% of the mothers of preterm infants return to work after their child is discharged from the hospital [33] ; this further leads to a decrease in their monthly household income. Families with a low income may encounter several barriers [34], that may harm parenting sense of competence and hinder the overall development of premature babies.

As expected, social support was positively associated with parenting sense of competence; this is in line with existing research [35]. A good social network can provide adequate informational, emotional, and physical assistance to parents of infants, whereas a poor social support can diminish parenting sense of competence by causing parents to feel inadequate in maintaining their roles and responsibilities [36]. In one study, parents were interviewed about their lived experiences of having a preterm infant; most expressed feeling of helpless and inadequate and wished for more information and support from professionals [37]. Therefore, healthcare providers should be fully aware of the diversity of preterm infants and their families and offer support initiatives at multiple levels (e.g., peer support and family-centered care) [38].

The current study has also added to the evidence that advanced parenting skills and parenting knowledge are significantly associated with a better parenting sense of competence among parents of premature infants. Previous empirical evidence has revealed a connection between parenting sense of competence and parenting knowledge and abilities [20]. Based on the social cognitive theory [39], parenting sense of competence can be fostered through four domains, namely: mastery experiences, vicarious experiences, verbal persuasion, and physiological/emotional arousal. Mastery of experiences encompasses parenting skills and knowledge. A high parenting sense of competence implies that parents know how to care for their premature infant, which in turn, requires them to have sufficient parenting knowledge and skills [7]. Donovan et al., found that insufficient knowledge of infant development was associated with reduced parenting sensitivity in mothers [40]. In another study, after parents gained adequate parenting experience, the level of parenting sense of competence followed an increasing trend [21]. Therefore, healthcare professionals should focus on parents with poor parenting knowledge and skills within the first year of a child’s birth, and offer professional guidance support, to help them cope with the difficulties of raising premature infants. This could be a rapid and efficient way of boosting parenting sense of competence.

This study also revealed that parents with more depressive symptoms tended to have a worse parenting sense of competence, this was supported by the findings of Ngai et al., who found that depression is a key factor affecting perceived maternal role competence and satisfaction postpartum [41]. Preterm birth puts parents in an unexpected situation; physical and mental fatigue from caring for a preterm infant, can lead to negative changes in the parents’ emotions and satisfaction [42]. Martinez-Torteya et al., noted that reducing postnatal depressive symptoms may be a crucial aspect of interventions aimed at improving parenting sense of competence [43]. Therefore, after preterm birth, healthcare providers should screen both fathers and mothers for depression and provide timely and targeted interventions if required.

Better family functioning implies that parents receive considerable family support [17]. In this study, parents with poorer family functioning tended to feel less competent and satisfied in their parenting role. However, although family functioning was significantly related to parenting sense of competence, it was not retained in the final regression model. A recent systematic review indicated that poor family functioning is strongly associated with depression [44], while another study revealed parental depression as a mediator between family functioning and parenting sense of competence [17]. Based on our own and the aforementioned findings, we suggest that instead of considering better family functioning as a factor for higher parental sense of competence, it may be wiser to look at the ways in which family functioning directly or indirectly affects parenting sense of competence among parents of preterm infants.

### Limitations

This study has several limitations. The participants comprised a convenience sample from one tertiary hospital in the Fujian Province (China). Therefore, the external validity of our findings may be limited. Furthermore, our study excluded preterm infants with specific diseases; thus, this population needs to be studied further in the future. The cross-sectional nature of this study precluded, causal inferences and precise mediation tests; to investigate causal relationships, additional studies with an experimental or longitudinal design are required. Finally, all the instruments used in this study were self-reported; thus, the risk of reporting bias cannot be ruled out.

## Conclusions

This study examined the level of parenting sense of competence and associated factors among Chinese parents of preterm infants. Parenting sense of competence was associated with parenting skills, parenting knowledge, social support and depression. This finding may support the establishment of constructive recommendations for healthcare professionals who clinically implement home-based prenatal and postnatal care. To improve parenting sense of competence among parents of preterm infants, interventional strategies can be adopted that enable the provision of comprehensive knowledge and, social support, to such parents and promote their psychological well-being.

## Data Availability

Datasets used and analyzed during this study are available from the corresponding author on reasonable request.

## Abbreviations

C-PSOC: Chinese version of the parenting sense of competence scale
PSCO-E: Perceived parental role competence subscale
PSCO-S: Parenting satisfaction subscale
EPDS: Edinburgh postnatal depression scale
SSRS: Social support rating scale
APGAR: Adaptation, partnership, growth, affection, and resolve
PICCS: Premature infant care competence scale for parents in transition
SD: Standard deviation

## References

[1] Kong X, Xu F, Wu R, Wu H, Ju R, Zhao X, Tong X, Lv H, Ding Y, Liu F (2016). Neonatal mortality and morbidity among infants between 24 to 31 complete weeks: a multicenter survey in China from 2013 to 2014. BMC Pediatr.

[2] Ream MA, Lehwald L. Neurologic Consequences of Preterm Birth. *Curr Neurol Neurosci Rep* 2018, 18(8):48.10.1007/s11910-018-0862-2. 10.1007/s11910-018-0862-2 29907917

[3] Neel MLM, Stark AR, Maitre NL (2018). Parenting style impacts cognitive and behavioural outcomes of former preterm infants: a systematic review. Child Care Health Dev.

[4] Studts CR, Pilar MR, Jacobs JA, Fitzgerald BK (2019). Fatigue and physical activity: potential modifiable contributors to parenting sense of competence. J Child Fam Stud.

[5] Latham RM, Mark KM, Oliver BR (2018). Coparenting and children’s disruptive behavior: interacting processes for parenting sense of competence. J Fam Psychol.

[6] Ngai FW, Wai-Chi Chan S, Holroyd E. Translation and validation of a chinese version of the parenting sense of competence scale in chinese mothers. *Nurs Res* 2007, 56(5):348-354.10.1097/01.NNR.0000289499.99542.94. 10.1097/01.NNR.0000289499.99542.94 17846556

[7] Baker B, McGrath JM, Pickler R, Jallo N, Cohen S. Competence and responsiveness in mothers of late preterm infants versus term infants. *J Obstet Gynecol Neonatal Nurs* 2013, 42(3):301-310.10.1111/1552-6909.12026. 10.1111/1552-6909.12026 PMC3774533 23601024

[8] Chung FF, Wan GH, Kuo SC, Lin KC, Liu HE. Mother-infant interaction quality and sense of parenting competence at six months postpartum for first-time mothers in Taiwan: a multiple time series design. *BMC Pregnancy Childbirth* 2018, 18(1):365.10.1186/s12884-018-1979-7. 10.1186/s12884-018-1979-7 PMC6127995 30189849

[9] Jandric S, Kurtovic A. Parenting Sense of Competence in Parents of Children With and Without Intellectual Disability. *Eur J Psychol* 2021, 17(2):75-91.10.5964/ejop.3771. 10.5964/ejop.3771 PMC8768480 35136430

[10] Gordo L, Oliver-Roig A, Martinez-Pampliega A, Iriarte Elejalde L, Fernandez-Alcantara M, Richart-Martinez M. Parental perception of child vulnerability and parental competence: The role of postnatal depression and parental stress in fathers and mothers. *PLoS One* 2018, 13(8):e0202894.10.1371/journal.pone.0202894. 10.1371/journal.pone.0202894 PMC6110487 30148877

[11] Messmer Uccelli M, Ponzio M. A case-control study assessing parenting sense of competence in people with multiple sclerosis. *Rehabil Psychol* 2018, 63(3):431-437.10.1037/rep0000211. 10.1037/rep0000211 30070560

[12] Garfield CF, Simon CD, Rutsohn J, Lee YS. Stress From the Neonatal Intensive Care Unit to Home: Paternal and Maternal Cortisol Rhythms in Parents of Premature Infants. *J Perinat Neonatal Nurs* 2018, 32(3):257-265.10.1097/jpn.0000000000000296. 10.1097/JPN.0000000000000296 PMC5976503 29194078

[13] Koliouli F, Gaudron CZ, Bourque CJ, Raynaud JP. Parental sense of competence, paternal stress and perceived construction of the relationship with the premature newborn: A mixed method study. *Early Hum Dev* 2022, 168:105576.10.1016/j.earlhumdev.2022.105576. 10.1016/j.earlhumdev.2022.105576 35483108

[14] Duan XF, Yang AP, Chen H. Social comparison tendency of mothers of preterm infants and its influence on parenting sense of competence. *Maternal and Child Health Care of China* 2018, 33(19):4501-4504.10.7620/zgfybj.j.issn.1001-4411.2018.19.60.

[15] Jing XBWL, Ming H, Li CP, Gao TT, Li HY (2018). Parenting sense of competence and self-efficacy among women with previous high-risk pregnancy:the influencing factors. J Nurs Sci.

[16] Shorey S, Ying L, Yobas P. Parenting Outcomes and Predictors of Parenting Satisfaction in the Early Postpartum Period. *West J Nurs Res* 2020, 43(1):13-24.10.1177/0193945920914593. 10.1177/0193945920914593 32389069

[17] Angley M, Divney A, Magriples U, Kershaw T. Social support, family functioning and parenting competence in adolescent parents. *Matern Child Health J* 2015, 19(1):67-73.10.1007/s10995-014-1496-x. 10.1007/s10995-014-1496-x PMC4233010 24833286

[18] Zhu Y, Zhou X, Yin X, Qiu L, Sun N, An R, Gong Y. Parenting sense of competence and its predictors among primiparous women: a longitudinal study in China. *BMC Pregnancy Childbirth* 2022, 22(1):548.10.1186/s12884-022-04881-y. 10.1186/s12884-022-04881-y PMC9260977 35799122

[19] Wu XL, Shi YX, Zhang YZ (2021). Influence of parenting sense of competence and mental resilience on postpartum depression in preterm primipara. Chin Nurs Res.

[20] Shorey S, Ng YPM, Ng ED, Siew AL, Morelius E, Yoong J, Gandhi M. Effectiveness of a Technology-Based Supportive Educational Parenting Program on Parental Outcomes (Part 1): Randomized Controlled Trial. *J Med Internet Res* 2019, 21(2):e10816.10.2196/10816. 10.2196/10816 PMC6391716 30758289

[21] Zachry AH, Jones T, Flick J, Richey P. The Early STEPS Pilot Study: The Impact of a Brief Consultation Session on Self-reported Parenting Satisfaction. *Matern Child Health J* 2021, 25(12):1923-1929.10.1007/s10995-021-03234-z. 10.1007/s10995-021-03234-z 34613553

[22] Cox JL, Holden JM, Sagovsky R. Detection of postnatal depression. Development of the 10-item Edinburgh Postnatal Depression Scale. *Br J Psychiatry* 1987, 150:782-786.10.1192/bjp.150.6.782. 10.1192/bjp.150.6.782 3651732

[23] Lau Y, Wang Y, Yin L, Chan KS, Guo X. Validation of the Mainland Chinese version of the Edinburgh Postnatal Depression Scale in Chengdu mothers. *Int J Nurs Stud* 2010, 47(9):1139-1151.10.1016/j.ijnurstu.2010.02.005. 10.1016/j.ijnurstu.2010.02.005 20219196

[24] Xiao SY (1994). Theoretical basis and application in research of Social Support rating scale. J Clin Psychiatry.

[25] Zheng J, Sun K, Aili S, Yang X, Gao L. Predictors of postpartum depression among Chinese mothers and fathers in the early postnatal period: A cross-sectional study. *Midwifery* 2022, 105:103233.10.1016/j.midw.2021.103233. 10.1016/j.midw.2021.103233 34968820

[26] Smilkstein G (1978). The family APGAR: a proposal for a family function test and its use by physicians. J Fam Pract.

[27] Huang Y, Liu Y, Wang Y, Liu D. Family function fully mediates the relationship between social support and perinatal depression in rural Southwest China. *BMC Psychiatry* 2021, 21(1):151.10.1186/s12888-021-03155-9. 10.1186/s12888-021-03155-9 PMC7953569 33711987

[28] Zhao XW. Study on the development and reliability and validity of Premature Infant Care Competency Scale (PICCS)for parents in transition. *Master* Shandong University; 2020.

[29] Yang X, Ke S, Gao LL. Social support, parental role competence and satisfaction among Chinese mothers and fathers in the early postpartum period: A cross-sectional study. *Women Birth* 2020, 33(3):e280-e285.10.1016/j.wombi.2019.06.009. 10.1016/j.wombi.2019.06.009 31235276

[30] Pennell C, Whittingham K, Boyd R, Sanders M, Colditz P. Prematurity and parental self-efficacy: the Preterm Parenting & Self-Efficacy Checklist. Infant Behav Dev, 35(4):678–88. 10.1016/j.infbeh.2012.07.009 22982267

[31] Perricone G, Morales MR, De Luca F, Carollo A, Maniscalco F, Caldas Luzeiro J, Polizzi C (2014). Coping and parental role competence of mothers of preterm infant. Minerva Pediatr.

[32] McLaurin KK, Hall CB, Jackson EA, Owens OV, Mahadevia PJ. Persistence of morbidity and cost differences between late-preterm and term infants during the first year of life. *Pediatrics* 2009, 123(2):653-659.10.1542/peds.2008 – 1439. 10.1542/peds.2008-1439 19171634

[33] Gennaro S. Leave and employment in families of preterm low birthweight infants. *Image J Nurs Sch* 1996, 28(3):193-198.10.1111/j.1547-5069.1996.tb00351.x. 10.1111/j.1547-5069.1996.tb00351.x 8854539

[34] Hall EM, Shahidullah JD, Lassen SR. Development of postpartum depression interventions for mothers of premature infants: a call to target low-SES NICU families. *J Perinatol* 2020, 40(1):1-9.10.1038/s41372-019-0473-z. 10.1038/s41372-019-0473-z 31439918

[35] Chavis L. Mothering and anxiety: Social support and competence as mitigating factors for first-time mothers. *Soc Work Health Care* 2016, 55(6):461-480.10.1080/00981389.2016.1170749. 10.1080/00981389.2016.1170749 27266719

[36] Lau R, Morse CA. Parents’ coping in the neonatal intensive care unit: a theoretical framework. *J Psychosom Obstet Gynaecol* 2001, 22(1):41-47.10.3109/01674820109049949. 10.3109/01674820109049949 11317609

[37] Lundqvist P, Weis J, Sivberg B. Parents’ journey caring for a preterm infant until discharge from hospital-based neonatal home care-A challenging process to cope with. *J Clin Nurs* 2019, 28(15–16):2966-2978.10.1111/jocn.14891. 10.1111/jocn.14891 31017322

[38] Hynan MT, Hall SL. Psychosocial program standards for NICU parents. *Journal of Perinatology* 2015, 35(1):S1-S4.10.1038/jp.2015.141. 10.1038/jp.2015.141 PMC4694191 26597799

[39] Bandura A. Social cognitive theory: an agentic perspective. *Annu Rev Psychol* 2001, 52:1-26.10.1146/annurev.psych.52.1.1. 10.1146/annurev.psych.52.1.1 11148297

[40] Donovan W, Taylor N, Leavitt L. Maternal self-efficacy, knowledge of infant development, sensory sensitivity, and maternal response during interaction. *Dev Psychol* 2007, 43(4):865-876.10.1037/0012-1649.43.4.865. 10.1037/0012-1649.43.4.865 17605520

[41] Ngai FW, Wai-Chi Chan S, Ip WY. Predictors and correlates of maternal role competence and satisfaction. *Nurs Res* 2010, 59(3):185-193.10.1097/NNR.0b013e3181dbb9ee. 10.1097/NNR.0b013e3181dbb9ee 20404775

[42] Loewenstein K, Barroso J, Phillips S. The Experiences of Parents in the Neonatal Intensive Care Unit: An Integrative Review of Qualitative Studies Within the Transactional Model of Stress and Coping. *J Perinat Neonatal Nurs* 2019, 33(4):340-349.10.1097/JPN.0000000000000436. 10.1097/JPN.0000000000000436 31651628

[43] Martinez-Torteya C, Katsonga-Phiri T, Rosenblum KL, Hamilton L, Muzik M. Postpartum depression and resilience predict parenting sense of competence in women with childhood maltreatment history. *Arch Womens Ment Health* 2018, 21(6):777-784.10.1007/s00737-018-0865-7. 10.1007/s00737-018-0865-7 PMC6240379 29860623

[44] Guerrero-Muñoz D, Salazar D, Constain V, Perez A, Pineda-Cañar CA, García-Perdomo HA. Association between Family Functionality and Depression: A Systematic Review and Meta-Analysis. *Korean J Fam Med* 2021, 42(2):172-180.10.4082/kjfm.19.0166. 10.4082/kjfm.19.0166 PMC8010447 32521579

